# Training set optimization of genomic prediction by means of EthAcc

**DOI:** 10.1371/journal.pone.0205629

**Published:** 2019-02-19

**Authors:** Brigitte Mangin, Renaud Rincent, Charles-Elie Rabier, Laurence Moreau, Ellen Goudemand-Dugue

**Affiliations:** 1 LIPM, Université de Toulouse, INRA, CNRS, Castanet-Tolosan, France; 2 GDEC, INRA, Clermont-Ferrand, France; 3 ISEM, Univ. Montpellier, CNRS, EPHE, IRD, Montpellier, France; 4 LIRMM, Univ. Montpellier, CNRS, Montpellier, France; 5 GQE-Le Moulon, INRA, Univ Paris-Sud, CNRS, AgroParisTech, Université Paris-Saclay, Gif-sur-Yvette, France; 6 LGB, Florimond Desprez Veuve & Fils, Cappelle-en-Pévèle, France; University of Texas School of Public Health, UNITED STATES

## Abstract

Genomic prediction is a useful tool for plant and animal breeding programs and is starting to be used to predict human diseases as well. A shortcoming that slows down the genomic selection deployment is that the accuracy of the prediction is not known a priori. We propose EthAcc (Estimated THeoretical ACCuracy) as a method for estimating the accuracy given a training set that is genotyped and phenotyped. EthAcc is based on a causal quantitative trait loci model estimated by a genome-wide association study. This estimated causal model is crucial; therefore, we compared different methods to find the one yielding the best EthAcc. The multilocus mixed model was found to perform the best. We compared EthAcc to accuracy estimators that can be derived via a mixed marker model. We showed that EthAcc is the only approach to correctly estimate the accuracy. Moreover, in case of a structured population, in accordance with the achieved accuracy, EthAcc showed that the biggest training set is not always better than a smaller and closer training set. We then performed training set optimization with EthAcc and compared it to CDmean. EthAcc outperformed CDmean on real datasets from sugar beet, maize, and wheat. Nonetheless, its performance was mainly due to the use of an optimal but inaccessible set as a start of the optimization algorithm. EthAcc’s precision and algorithm issues prevent it from reaching a good training set with a random start. Despite this drawback, we demonstrated that a substantial gain in accuracy can be obtained by performing training set optimization.

## Introduction

Prediction of unobserved individuals using genomic information has gained increasing importance in plant and animal breeding [1, 2]. Moreover, it is an accurate tool for prediction of complex diseases in humans [3, 4] and is included in the precision medicine initiative [5].

Basically, a training set of individuals, the so-called training set, that is both phenotyped and genotyped is used to train a model that is applied to predict unobserved individuals, the so-called test set, on the basis of only genotyping data from the latter. Many methods have been proposed for the training model step that are included in the mixed model framework [6, 7], in penalized regression methods [8], in Bayesian modeling [9], and in semiparametric and nonparametric learners [10, 11]. These methods have been comprehensively compared, and depending on the trait under study, one or another method has been shown to be more reliable, but the best performers provide comparable accuracy rates [12, 13]. In any case, genomic best linear unbiased prediction (GBLUP) [7] was shown to be competitive with more complicated models. Its ease of use and its efficient computer implementation explain its development into a reference model. Moreover, the flexibility of the mixed model framework for modeling of nonadditive genetic factors or for incorporation of functional knowledge [14] explains the large number of extensions of the GBLUP model.

A shortcoming that slows down the genomic selection deployment is that the accuracy of the prediction is not known a priori and depends on a number of factors such as the size of the training set, trait architecture (especially its heritability), density of markers, relatedness between tests and trainings, and others. The prediction accuracy is useful for at least two purposes. One of them is to decide whether genomic selection is worth applying to a crop for a trait of interest. This accuracy is defined as the expectation of the prediction accuracy for a training set randomly drawn in a population. The second purpose is to allow for optimization of the training set used to predict the test individuals and must involve a quantity that enables discrimination among several training sets, and therefore it must be defined given a training set.

Most of the accuracy proxies have been developed for the first purpose, that is the expectation of prediction accuracy for a random training set belonging to a population. Moreover, they have been developed by means of predictions obtained in a linear mixed model, known as GBLUP or equivalently ridge regression best linear unbiased prediction (RR-BLUP) because there is an analytical prediction of the genetic value in this case. Nonetheless, the first proxy proposed by Daetwyler et al. [15] was developed within a simpler model assuming that the genetic value is explained by a fixed number of independent quantitative trait loci (QTLs). This seminal formula was generalized later by Goddard [16] who linked accuracy to the effective number of segments in the genome by adding the assumption that the QTL effects are independent and identically distributed. From these two papers, different formulas have been derived that linked the prediction accuracy to heritability *h*^2^, training population size *T*, the number of markers *M*, and the number of effective segments in the genome *M*_*e*_ [16–18]. Some authors [19] compared these formulas with a meta-analysis of 13 papers in which prediction accuracy was computed using both simulated or real data. They showed that training population size *T* and the effective number of segments *M*_*e*_ have a significant impact on the accuracy; this situation is cause for concern because *M*_*e*_ can be estimated in several models that yield very different values [20]. They also demonstrated that among the different formulas, none outperforms the others. Until recently, Goddard’s formula has continued to give rise to new works such as the improvement proposed in [21] for the *M*_*e*_ parameter. Nevertheless, all these accuracy proxies derived from Goddard’s approach are based on a number of statistical and mathematical approximations that were clearly explained in [22]. That paper highlighted the difficulties of finding a proxy that reflects correctly the diversity of situations of training population structures and linkage disequilibrium between markers and QTLs.

Another proxy came from Rabier et al. [20] who developed a theoretical formula of the accuracy conditional to a given training set. In contrast to Goddard, Rabier et al [20] did not assume the independence of the QTLs or a distribution of the QTL effects but attempted to derive their formula using a fixed number of known causal QTLs. The major drawback of this theoretical formula is that the QTL locations and effects have to be estimated first and plugged into the formula to get an estimate of the prediction accuracy, that we call “EthAcc” here (Estimated THeoretical ACCuracy). Contrary to the above proxies that are estimates of the expectation of prediction accuracy for a training set randomly drawn from a given population, EthAcc is an estimator of the genomic prediction accuracy of a given training set. It could then be used to optimize the training set to increase the accuracy by choosing the trainings that together performed the best on the test predictions.

Panel optimization for genomic prediction was first proposed by Rincent et al [23]. They supposed that a panel of candidates has been genotyped and that the goal is to choose the best set to phenotype (i.e., the training individuals). The same objective was later addressed by the authors of [24]. Both research groups proposed to perform panel optimization with a fixed size of the training set, which is consistent when assuming a limited budget proportional to candidates to be phenotyped (i.e., the training set). Moreover, the two criteria upon which they based the optimization generally increase with the training set size, and therefore a fixed size is necessary to obtain an optimized training set that is smaller than the whole panel of candidates. The question of training panel optimization for the test prediction is more suitable for genomic selection per se. In a breeding company, as an example, past breeding panels that have already been phenotyped are nowadays genotyped. They constitute the resources upon which genomic models can be trained. At the same time, new breeding resources are created and genotyped but are still not phenotyped. These resources have to be subjected to genomic selection, and the key task is to choose the best training set from genotyped and phenotyped past resources to optimally predict the current resources.

## Materials and methods

The genomic prediction of the genetic value of test individuals was based on GBLUP [7], and we define the accuracy of genomic prediction as Pearson’s correlation between its phenotype and its BLUP-value for a random test individual and a given training set. We refer to this correlation as the accuracy in the text below.

This accuracy is a theoretical quantity that is usually estimated using the mean of empirical Pearson’s correlation between phenotypic values and their GBLUP for a random test set. In statistics, this estimate is called an empirical estimate or sample estimate, but we call it the test set (TS) accuracy in the analysis that follows. This TS accuracy can be calculated only if phenotypes are observed in test individuals, which is the case when the TS is randomly drawn within a dataset or obtained by a simulation process. This TS accuracy was compared to three methods that estimate the accuracy without making use of the test set phenotypic observations.

### Accuracy estimated given a training set

The idea behind finding an estimate of the accuracy is to confound the causal-QTL model which emulates the genetic value with QTLs to the model used to make the prediction of the genetic value using markers. As we focus on the prediction obtained by GBLUP or equivalently by RR-BLUP, this confusion means that each marker is assumed to be a QTL, and the QTL effects are assumed to be independent and identically distributed according to a Gaussian distribution. We called this confusion “the GBLUP model of the genetic value”. In the GBLUP model, the coefficient of determination (CD) of the genetic value of a test individual is by definition the square correlation between the genetic value and its predictor. This accuracy is known to be linked to the accuracy involving the phenotypic value by the square root of heritability [25]; thus, the first accuracy estimate is

$$\begin{array}{c} {\hat{\rho }}^{\text{CD}}=\frac{1}{n_{\text{test}}}\sum_{i}\sqrt{\frac{\text{Var}(u_{\text{test},i})}{\text{Var}\left(u_{\text{test},i}\right)+\sigma_{\varepsilon }^{2}}\text{CD}\left(u_{\text{test},i}\right)} \end{array}$$

where *u*_test,*i*_ is the random genetic value of the *i*th individual in a TS, *n*_test_ is the number of test individuals, and $\sigma_{\varepsilon }^{2}$ denotes the residual variance using the GBLUP model.

The second method for predicting the accuracy is based on the prediction error variance (PEV) involving the GBLUP model. By definition, PEV is the variance of the difference between an individual genetic value and its predictor. In the GBLUP model, it can be proved that $\text{Cov}({\hat{u}}_{\text{test},i}^{BLUP},u_{\text{test},\text{i}})=\text{Var}({\hat{u}}_{\text{test},i}^{BLUP})$ and so $\text{PEV}(u_{\text{test},\text{i}})=\text{Var}(u_{\text{test},i})-\text{Cov}({\hat{u}}_{\text{test},i}^{BLUP},u_{\text{test},\text{i}})$, where ${\hat{u}}_{\text{test},i}^{BLUP}$ is the BLUP predictor of the *i*th individual. Therefore, the second accuracy estimate is

$$\begin{array}{c} {\hat{\rho }}^{\text{PEV}}=\frac{1}{n_{\text{test}}}\sum_{i}\sqrt{\frac{\text{Var}(u_{\text{test},i})}{\text{Var}\left(u_{\text{test},i}\right)+\sigma_{\varepsilon }^{2}}\left(1-\frac{\text{PEV}(u_{\text{test},i})}{\text{Var}(u_{\text{test},i})}\right)} \end{array}$$

Both CD and PEV rely on the GBLUP model, or equivalently the RR-BLUP model [26] that is presented here:

$$\begin{array}{c} y_{\text{train}}=1\mu +X_{\text{train}}\beta +\varepsilon \end{array}$$

where *μ* is the global mean, **1** represents a vector of 1, ***X***_train_ is the matrix of SNP genotypes for individuals in the training set, and ***y***_train_ denotes their observed phenotypes. ***β*** is the vector of SNP effects assumed to be independent and identically distributed (iid) with Gaussian distribution $N(0,\sigma_{\beta }^{2})$, and ***ε*** is the vector of residual errors assumed to be iid with Gaussian distribution $N(0,\sigma_{\varepsilon }^{2})$, independent of ***β***. Moreover, *u*_test,*i*_ is assumed to be equal to $x_{\text{test},i}^{\prime }\beta$, where $x_{\text{test},i}^{\prime }$ is the line vector of SNP genotypes for test individual *i*; accordingly, we get $\text{Var}(u_{\text{test},i})=\sigma_{\beta }^{2}x_{\text{test},i}x_{\text{test},i}^{\prime }$.

The third method is based on the work of [20] who theoretically derived the accuracy when QTL locations and effects are known. They developed their formula within a framework where the RR-BLUP model serves as an instrumental approach to get ***β*** estimated values, whereas a linear causal model with a fixed number of known QTLs is assumed to model the phenotype as follows:

$$\begin{array}{c} y_{\text{train}}=1\mu +Q_{\text{train}}\theta +e \end{array}$$

where ***Q***_train_ is the matrix of QTL genotypes for individuals in the training set, ***θ*** represents the vector of QTL effects, and ***e*** denotes the vector of residual errors assumed to be iid with Gaussian distribution $N(0,\sigma_{e}^{2})$. Genetic value *g*_test,*i*_ is assumed to be equal to $q_{\text{test},i}^{\prime }\theta$, where $q_{\text{test},i}^{\prime }$ is the line vector of QTL genotypes for the *i*th test individual.

Using the instrumental RR-BLUP model and the causal-QTL model, they obtained the following theoretical formula of the accuracy for an individual randomly sampled in the test population:

$$\begin{array}{c} \rho =\frac{\theta^{\prime }\;E(q_{\text{test},i}{x_{\text{test},i}}^{\prime })X_{\text{train}}^{\prime }H^{-1}Q_{\text{train}}\theta }{{\left(\sigma_{e}^{2}\;E({∥x_{\text{test},i}X_{\text{train}}^{\prime }H^{-1}∥}^{2})\;+\;\theta^{\prime }Q_{\text{train}}^{\prime }H^{-1}X_{\text{train}}\text{Var}(x_{\text{test},i})X_{\text{train}}^{\prime }H^{-1}Q_{\text{train}}\theta \right)}^{1/2}{\left(\sigma_{g}^{2}+\sigma_{e}^{2}\right)}^{1/2}} \end{array}$$

where ‖.‖ is the *L*^2^ norm, $\sigma_{e}^{2}$ represents the error variance in the causal model, $\sigma_{g}^{2}=\text{Var}(g_{\text{test},i})$ and $H=\left(X_{\text{train}}X_{\text{train}}^{\prime }+\frac{\sigma_{\varepsilon }^{2}}{\sigma_{\beta }^{2}}I\right)$, with ***I*** denoting the identity matrix. Note that the formula is correct if matrices of SNP genotypes are previously column centered to make them orthogonal to *μ*.

To estimate *ρ*, all quantities depending on a random individual in the test set are replaced by the empirical or sample estimate (i.e., the mean across test individuals). $\sigma_{e}^{2}$ is estimated by the least square method in the causal-QTL model by means of genotypes and phenotypes of training set individuals. Nevertheless, the location of QTLs and their effect have to be estimated if they are unknown.

For the three accuracy estimates, variance parameters $\sigma_{\varepsilon }^{2}$ and $\sigma_{\beta }^{2}$ are estimated by restricted maximum likelihood (REML) using genotypic and phenotypic data of training set individuals.

### Estimation of a causal-QTL model

The authors of [27] used penalized regression methods to detect the QTLs and to estimate their effects though it is well known that penalized regression yields biased estimators of QTL effects. In this paper, we used a two-step procedure, the first step was to locate the QTLs via multi-QTL methods developed for a genome-wide association study (GWAS), then the QTL effects were estimated via a classical linear model by the ordinary least square method as well as the error variance in the causal model $\sigma_{e}^{2}$.

One of multi-QTL methods is the forward selection approach of the multilocus mixed model (MLMM) proposed in [28]. A classical marker-by-marker GWAS model [29] with a VanRaden’s kinship matrix [7] for the polygenic effect and no fixed structure effect is employed. The forward selection approach is an iterative technique, where at each step, the SNP with the minimum p-value is added into the model as a fixed effect, and a rescan of the remaining SNPs is performed. The iterative procedure stops when the variance of the polygenic effect is close to zero, meaning that the discovered SNPs that have been added into the model as fixed effects together explain almost all the polygenic variability. This final model outputs the discovered SNPs as causal QTLs.

The others multi-QTL methods used penalized regressions. They were thoroughly compared for GWAS by Waldman et al. [30]. Consequently, we based our choices for penalization parameters of the least absolute shrinkage and selection operator (LASSO) and the elastic net (EN) upon their comparisons. We computed LASSO shrinkage parameters λ by a 10-fold cross-validation schema, and we calculated the optimal λ from the minimum mean square error (minMSE) of the predictor and minMSE plus one standard error (1SE) of minMSE. Recently, Yi et al. [31] proposed a false discovery rate (FDR) control to make the choice of the λ parameter. We chose the analytical FDR control because it has been shown to perform the best on SNP selection [31]. The EN *α* parameter was set to 0.5 and 0.1. To complete the penalized regression methods, we added the adaptive LASSO [32] that gives the most accurate results when QTL effects are estimated by penalized regression estimators [27].

Once causal QTLs were located, their effects were all together estimated by the ordinary least square method via a classical linear model, as well as the error variance of this estimated causal-QTL model. The ordinary least square method was applicable because we limited the number of QTLs searched in function of the number of individuals. Nevertheless, in practice, this constraint on the number of QTLs has never been applied.

### Panel optimization

Panel optimization consists of choosing (within a panel of candidates) the set of training individuals that better predicts a test belonging to a population of tests. One research group [23] proposed to use the CDmean criterion to make the choice. Later, [24] suggested to perform the optimization on the basis of PEV. CDmean is the mean across *n*_test_ test individuals *i* (i.e., sample estimate) of the CD for contrast between individual genetic value *u*_test,*i*_ and the population mean (test + training individuals). The criterion on the basis of PEV is the mean across *n*_test_ test individuals *i* (i.e., sample estimeate) of PEV of individual genetic value *u*_test,*i*_. CDmean served as the reference for comparison with EthAcc because it has been shown to perform slightly better than PEV on panel optimization [23] even for a mildly structured population [33, 34].

The panel optimization burden was dealt with by the hill-climbing algorithm with exchange moves that was proposed in [23]. We did not implement a stopping criterion to always perform a given number of exchange moves. The number of exchange moves was set to 5000.

The starting training set of the hill-climbing algorithm for both criteria was the optimal training set found by maximization of the TS accuracy, i.e., we found the training set that gave a maximum for the mean correlation between GBLUP and the observed phenotypes of the test individuals. This maximization of the TS accuracy was possible because the TS was randomly drawn within the whole dataset, and therefore test individuals had phenotypes. Having this set as a start of the algorithm prevents it from getting stuck in a local maximum that is far from the maximum of the TS accuracy. Starting with this optimal training set, we were able to measure how the different criteria will degrade the maximum of the TS accuracy, in other words, how big the decrease in accuracy (caused by each criterion) will be.

**Fig 1:**
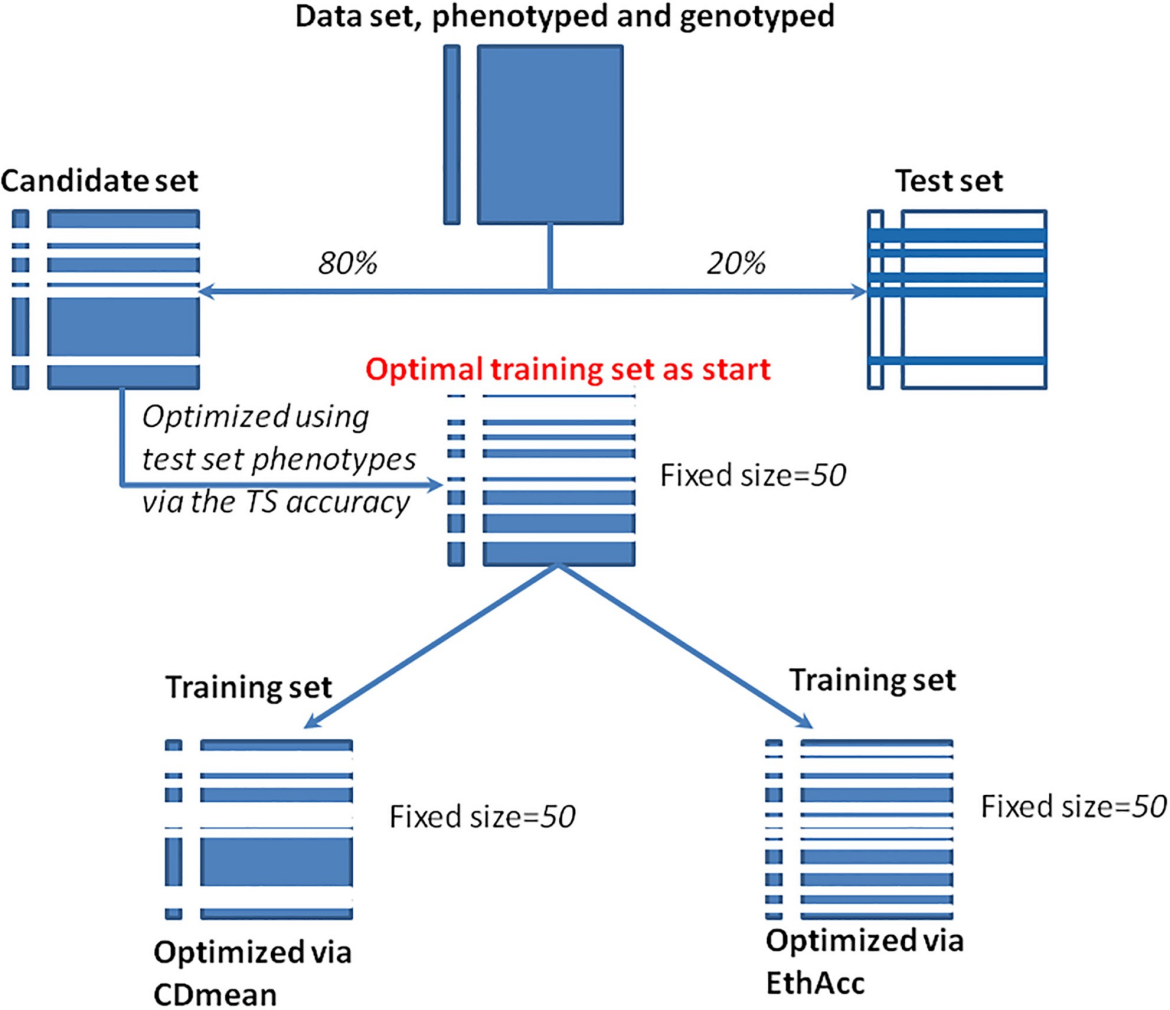
Illustration of the sampling and of the optimization process that were implemented to compare CDmean and EthAcc criteria.

Fig 1 resumes the optimization process for a random sampling of the test set.

For CDmean calculation, the variance components of the RR-BLUP model, $\sigma_{\varepsilon }^{2}$ and $\sigma_{\beta }^{2}$, were estimated by REML with the entire candidate panel to obtain the most precise estimated values. The causal-QTL model of EthAcc was discovered by the forward selection approach of MLMM applied to the current training set. It was thus re-evaluated at each new training set.

At the end of the optimization burden, the TS accuracy was computed via the RR-BLUP model trained on the respective optimal training set of each criterion.

### Simulation implementations

Method comparisons were made by randomly drawing a TS within the whole panel. Unless precise, 20% of the panel served as a TS and the remaining individuals were the candidates or the largest training set when we did not perform training set optimization. To ensure accurate comparisons, the TS accuracy and the accuracy estimates were computed on the same TS. The results were obtained for 30 random TS draws.

Predicted genetic values of TS individuals were obtained by estimating SNP effects with the training set by means of the mixed.solve function of R package rrBLUP https://cran.r-project.org/web/packages/rrBLUP/index.html. This function was also applied to compute CD, PEV, and CDmean via our own R code.

The R code for computing EthAcc (from a training set that is genotyped and phenotyped and a test set that is only genotyped) is given in S1 File. We used our own R code, which was rewritten based on Segura’s code [28] and that is now available on CRAN https://cran.r-project.org/web/packages/mlmm.gwas/index.html, to implement the forward selection MLMM approach. The glmnet R package https://cran.r-project.org/web/packages/glmnet/index.html was used to perform localization of QTLs by penalized regression methods. The parcor R package https://cran.r-project.org/web/packages/parcor/index.html with the parameter of cross-validation set to 5 was used to perform adaptive LASSO.

To avoid computation issues, SNP filtration on minor allele frequency (MAF) was carried out. All code can be provided on request.

### Plant material

Maize data has been downloaded from http://www.genetics.org/content/suppl/2012/08/03/genetics.112.141473.DC1. Sugar beet, sunflower and wheat data are available at https://lipm-browsers.toulouse.inra.fr/pub/Mangin2018-PlosOne/

#### Sugar beet

A panel of 2101 elite lines of diploid sugar beet (*Beta vulgaris* L.), which resulted from many different crosses in Florimond Desprez’s breeding program, was analyzed. Testcross progenies of these lines were evaluated in unbalanced multienvironment trials for seven traits: the white sugar yield (WSY, t/ha), sugar content (S, %), white sugar content (WS, %), the root yield (RY, t/ha), potassium content (K, meq/100 g), sodium content (Na, meq/100 g), and *α*-amino nitrogen content (N, meq/100 g). The sugar beet panel was fingerprinted with 836 SNP markers, but the panel was analyzed with 692 SNPs after filtration for MAF and missing data (see details in S1 File). The structure of subpopulations in this panel was then studied. We applied hierarchical clustering to principal components using the FactoMineR package https://cran.r-project.org/web/packages/FactoMineR/index.html [35] in R software to assign each individual to a subpopulation after principal component analysis (PCA). The HCPC function of the FactoMineR package implements this calculation after having constructed the hierarchy and suggests an optimal level for division.

#### Sunflower

Sunflower data covered hybrids obtained by crossing 36 restorer of CMS PET1 male sterility lines serving as males to 36 maintainer lines as females in an incomplete factorial as described elsewhere [36]. Hybrid genotyping data were obtained from the whole-genome sequencing of their parents. A total of 468 194 nonredundant SNPs passed the different filters [36] and were used to compute their kinships. The phenotype trait, adjusted for field effects [36], was oil content in the 13EX01 environment with nonmissing data on 272 hybrids.

The test set was compiled by randomly sampling seven parents among the 72 parents (roughly 10%), and we placed all their descendants in the test set. This sampling produced a test set consisting of only T1 or T0 hybrids, as described by authors in [37], which have been shown to be difficult to predict because they are more distant from the possible training sets [36].

#### Maize

The two maize diversity panels employed in this study are some lines of the Flint and Dent panels that passed the genotyping and phenotyping filters described in [23]. Each panel is composed of 261 lines, genotyped for 30 027 and 29 094 markers for the Dent and the Flint lines, respectively, and crossed to a tester belonging to the other panel for hybrid phenotyping. Two traits were analyzed: the mean of male flowering time (Tass_GDD6) and the plant dry matter yield (DM_Yield) that were observed in 2010 at four or five locations in Europe.

#### Wheat

A sample of 296 bread wheat accessions was employed for this study. This sample is a part of the INRA bread wheat core collection of 372 accessions (372CC) set up by [38]. Each accession was genotyped for 2013 markers (SSR, DArT, and SNP) covering the whole genome [39, 40]. The SSR markers were transformed into biallelic markers by considering the different alleles independently. Each accession was phenotyped for heading date (day of year, DOY) in Clermont-Ferrand (France) in 2005. For this purpose, 10 seeds of each accession were sown in a single row on 27 October 2004. Ear emergence day of the main tiller of five to six individual plants was recorded when half of the ear had emerged from the flag leaf. The numbers were averaged to obtain the heading date for each accession [40].

## Results

### EthAcc as an accurate estimate of the accuracy

The theoretical accuracy formula proposed by Rabier et al [20] was shown by simulation to be an accurate predictor of the accuracy when causal-QTLs are known. Nevertheless, the QTL estimation step may have destroyed this desirable property. Thus, we studied the ability of EthAcc to predict the accuracy.

#### How to estimate the causal QTLs for EthAcc?

The question of the best choice of the QTL detection methods necessary for EthAcc computation was addressed first. Method comparisons were made by randomly drawing a TS within the whole sugar beet panel. The mean accuracy and mean square error (MSE) as compared to the TS accuracy, were computed based on 100 random TSs for each trait. All traits were well predicted with the mean accuracy ranging from 0.59 for *α*-amino nitrogen content (N) to 0.76 for sugar content (S), white sugar content (WS), and sodium content (Na). The most important trait for breeding, the white sugar yield (WSY), was predicted with the accuracy 0.60 (Table 1). Results clearly highlighted MLMM forward selection as the best choice for locating causal QTLs to be plugged into EthAcc. Whatever the trait that was considered, the MLMM QTL search outperformed all the other methods tested (lowest MSE). The influence of the criterion used to choose the LASSO shrinkage parameter seems to be the most important factor in the ranking of penalized regression methods. After the best performer (MLMM), the penalized regression methods are classified by, first, the criterion of minimum MSE plus one standard error (Lasso.1se, EN05.1se, EN01.1se), then the minimum MSE criterion (Lasso.min), and finally the FDR criterion (EN05.FDR). The adaptive LASSO was ranked between penalized regresions using the best criterion (.1se) and the second one (.min). For the WSY trait (which is one of the most difficult traits to predict) having the accuracy of 0.60 on average, the MLMM method reached the lowest MSE (2.13 10^−3^), whereas the EN05.FDR method was fivefold worse, with the highest MSE (1.09 10^−2^).

**Table 1:** The accuracy and MSE of EthAcc compared to the TS accuracy according to several methods for estimation of causal QTLs on different traits (mean over 100 random test-training sets).

Trait^a^	Mean accuracy	Mean Square Error						
		MLMM	Lasso.min^b^	Lasso.1se	EN05.1se^c^	EN01.1se	EN05.FDR	adp.Lasso^d^
K	0.74	6.70 10^−4^	1.77 10^−3^	9.28 10^−4^	8.84 10^−4^	8.76 10^−4^	4.19 10^−3^	1.37 10^−3^
Na	0.76	4.86 10^−4^	1.41 10^−3^	5.96 10^−4^	5.75 10^−4^	6.59 10^−4^	3.67 10^−3^	9.72 10^−4^
N	0.59	1.50 10^−3^	5.37 10^−3^	2.68 10^−3^	2.74 10^−3^	2.90 10^−3^	1.60 10^−2^	3.94 10^−3^
S	0.76	6.28 10^−4^	1.36 10^−3^	7.31 10^−4^	6.99 10^−4^	8.56 10^−4^	7.20 10^−3^	1.06 10^−3^
WS	0.76	5.35 10^−4^	1.51 10^−3^	7.03 10^−4^	7.56 10^−4^	1.04 10^−3^	5.62 10^−3^	1.14 10^−3^
RY	0.72	9.56 10^−4^	2.24 10^−3^	1.06 10^−3^	1.05 10^−3^	1.02 10^−3^	1.05 10^−2^	1.69 10^−3^
WSY	0.60	2.13 10^−3^	5.43 10^−3^	2.48 10^−3^	2.24 10^−3^	2.28 10^−3^	1.09 10^−2^	3.96 10^−3^
Mean	0.70	9.86 10^−4^	2.73 10^−3^	1.31 10^−3^	1.28 10^−3^	1.38 10^−3^	8.28 10^−3^	2.02 10^−3^

^*a*^ potassium content in meq/100 g (K), sodium content in meq/100 g (Na), *α*-amino nitrogen content in meq/100 g (N), sugar content in % (S), white sugar content in % (WS), the root yield in t/ha (RY), and the white sugar yield in t/ha (WSY)

^*b*^ .min, .1se, and .FDR: choice of the LASSO shrinkage parameters λ based on the minimum MSE of the predictor (minMSE), minMSE plus one standard error (1SE) of minMSE and FDR, respectively

^*c*^ ENx: EN with its *α* parameter equal to x,

^*d*^ adp.Lasso: adaptive LASSO,

#### EthAcc behavior shows that “bigger is not always better”

The structure of the sugar beet panel was analyzed to assess the influence of population structure on the accuracy of prediction. The optimal cluster number of the hierarchical clustering in PCA [35] was set to 2. Sugar beet lines were represented in the first two principal components (see Fig A in S1 File). Clusters contained respectively 676 (Panel_A) and 1425 lines (Panel_B).

We addressed the question of whether it is better to train a model within a cluster or by means of the two clusters for prediction of a TS belonging to a unique cluster. We also compared EthAcc to the two other estimators of the accuracy: CD and PEV. TSs were randomly drawn specifically in a unique cluster (20% of the cluster size), and the model was trained either with all the remaining individuals of this cluster or with the remaining individuals of the whole panel (i.e., both clusters). Results on the mean accuracy among 100 randomly drawn TSs together with EthAcc, CD, and PEV means for each trait are presented in Table 2. When the TS belonged to Panel_A, it was better to include in the training set only the remaining individuals of Panel_A (i.e., 540 individuals) rather than adding all individuals of Panel_B (i.e., 1425 individuals). Indeed, whatever the trait analyzed, the mean accuracy was higher when both trainings and tests belonged to the same cluster, even if training sets were smaller (540 vs 1965 individuals). The increase in accuracy reached 14% (*α*-amino nitrogen content) and was of 5% on average among all the traits, while the training set was more than threefold smaller. Opposite results were obtained in the other cluster (Panel_B). In this case, the mean accuracy was always higher when all the remaining individuals from both clusters were used. The increase in the training set size (1140 vs 1816 individuals) led to an 8% increase in the accuracy on average among all the traits. The largest increase, 19%, was obtained for WSY with the mean accuracy of 0.640 when the training set was composed of individuals belonging to both clusters, whereas the mean accuracy was only of 0.536 when the training set was composed only of individuals belonging to Panel_B.

**Table 2:** The accuracy and its values estimated by EthAcc, CD, and PEV using sugar beet structures in two clusters (Panel_A and Panel_B) on several traits (the mean for 100 random test sets).

Trait^a^	Test set^b^	Training set^b^	Mean accuracy	Estimated by		
				EthAcc	CD	PEV
K	Panel_A	Panel_A+B	0.712	0.696	0.818	0.818
K	Panel_A	Panel_A	0.742	0.734	0.823	0.824
Na	Panel_A	Panel_A+B	0.660	0.675	0.815	0.815
Na	Panel_A	Panel_A	0.689	0.676	0.776	0.777
N	Panel_A	Panel_A+B	0.500	0.552	0.744	0.745
N	Panel_A	Panel_A	0.571	0.588	0.678	0.680
S	Panel_A	Panel_A+B	0.665	0.684	0.851	0.851
S	Panel_A	Panel_A	0.682	0.690	0.801	0.802
WS	Panel_A	Panel_A+B	0.680	0.691	0.851	0.851
WS	Panel_A	Panel_A	0.700	0.698	0.809	0.810
RY	Panel_A	Panel_A+B	0.654	0.655	0.826	0.827
RY	Panel_A	Panel_A	0.688	0.699	0.790	0.792
WSY	Panel_A	Panel_A+B	0.583	0.562	0.735	0.735
WSY	Panel_A	Panel_A	0.597	0.603	0.702	0.703
K	Panel_B	Panel_A+B	0.752	0.780	0.887	0.888
K	Panel_B	Panel_B	0.714	0.715	0.788	0.789
Na	Panel_B	Panel_A+B	0.802	0.814	0.895	0.895
Na	Panel_B	Panel_B	0.773	0.769	0.814	0.815
N	Panel_B	Panel_A+B	0.637	0.669	0.869	0.869
N	Panel_B	Panel_B	0.567	0.560	0.733	0.734
S	Panel_B	Panel_A+B	0.800	0.813	0.899	0.900
S	Panel_B	Panel_B	0.759	0.757	0.817	0.818
WS	Panel_B	Panel_A+B	0.798	0.813	0.902	0.903
WS	Panel_B	Panel_B	0.759	0.754	0.820	0.822
RY	Panel_B	Panel_A+B	0.750	0.779	0.887	0.888
RY	Panel_B	Panel_B	0.703	0.696	0.783	0.785
WSY	Panel_B	Panel_A+B	0.640	0.644	0.865	0.865
WSY	Panel_B	Panel_B	0.536	0.527	0.678	0.680

^*a*^ potassium content in meq/100 g (K), sodium content in meq/100 g (Na), *α*-amino nitrogen content in meq/100 g (N), sugar content in % (S), white sugar content in % (WS), the root yield in t/ha (RY), and the white sugar yield in t/ha (WSY)

^*b*^ cluster(s) to which the individual belongs

The comparison of the three estimators of the accuracy highlighted the correct behavior of EthAcc and showed that both CD and PEV are not accurate. The mean accuracy was correctly estimated by EthAcc, whereas CD and PEV were very close and generally overestimated the mean accuracy. Contrary to CD and PEV, EthAcc was the only estimator showing that the bigger training set was not always better for all the traits; this result was consistent with the mean accuracy. We performed a mean test comparison of the three estimators for their ability to evaluate the TS accuracy via the standard error correction proposed by [41] to take into account the dependencies of the sampled TSs (see the standard error correction and the test p-values in Table A in S1 File). Accuracy values estimated by CD and PEV were found to be significantly different (5% error risk) from the TS accuracy for all the traits and all the test-training panels (min p-value of 10^−21^, max p-value of 4 10^−2^), whereas the accuracy estimated by EthAcc was never deemed significantly different (min p-value of 6 10^−2^, max p-value of 0.99). The worse case for EthAcc, i.e., where EthAcc and the TS accuracy showed the largest difference, involved the root yield (RY) trait, a test set in Panel_B, and a training set belonging to both clusters. Nonetheless, on average among all the traits and all the test-training configurations, the accuracy estimated by EthAcc and that obtained on average seemed very close (mean p-value of 0.64).

**Fig 2:**
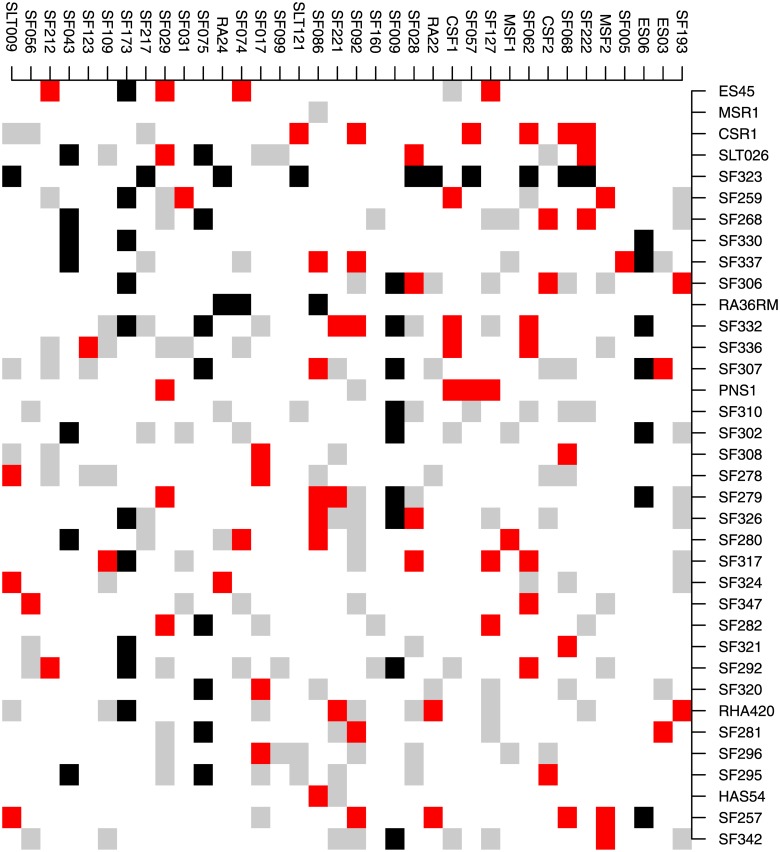
SUNRISE hybrids obtained from 36 maintainer lines (females, rows) crossed to 36 restorer lines (males, columns). Parental lines are arranged according to the hierarchical clustering based on VanRaden’s kinship matrices. Black squares indicate the 54 T0 or T1 hybrids in the test set, red squares are the 77 training individuals, and grey squares are hybrids that were among candidates but were not chosen to be in the training set.

We showed that on average, the biggest training set is not always the best to make a prediction in a structured population. This conclusion is also valid for a test set in a nonstructured population, as is the case for the sunflower hybrid population. Fig 2 presents a test set of T1 or T0 hybrids having a training set of 77 hybrids that yielded a TS accuracy of 0.745, whereas the TS accuracy with all the 218 hybrids as trainings is 0.722. This is not a great difference, but it indicates that in a particularly homogenous population that has not evolved, a training set adapted to the tests to be predicted can perform better than a bigger panel.

### Comparison of EthAcc and CDmean for training set optimization

**Fig 3:**
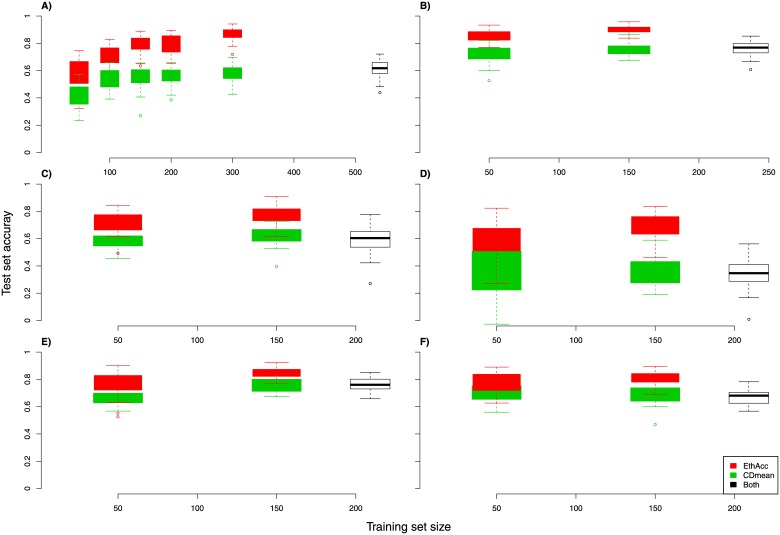
A significant increase in the TS accuracy produced by training set optimization with EthAcc compared to CDmean for several training set sizes and different datasets. The optimal (but inaccessible without test phenotypes) training set is the starting set of the optimization process. The black boxplot corresponds to the largest training set. A) WSY within sugar beet Panel_A. B) DOY for wheat. C) DM_Yield within the Dent panel. D) DM_Yield within the Flint panel. E) Tass_GDD6 within the Dent panel. E) Tass_GDD6 within the Flint panel. Boxplots were constructed from 30 random test sets representing 20% of the whole panel.

Fig 3 depicts the comparison of the accuracy for optimization based on EthAcc with that based on CDmean. It is worth remembering that what is shown in this figure is the degradation produced by CDmean and EthAcc of the maximum of the TS accuracy because the starting point of the hill-climbing algorithm for the two criteria is the training set giving the maximum of the TS accuracy. In all the cases, EthAcc yielded a smaller decrease in prediction accuracy than CDmean did. This result is expected because EthAcc estimates the accuracy via a model with a fixed number of QTLs whereas CDmean estimates the accuracy by means of a model with as many QTLs as markers, moreover assuming that QTL effects are Gaussian distributed.

### Toward an understanding of differences between EthAcc and CDmean

**Fig 4:**
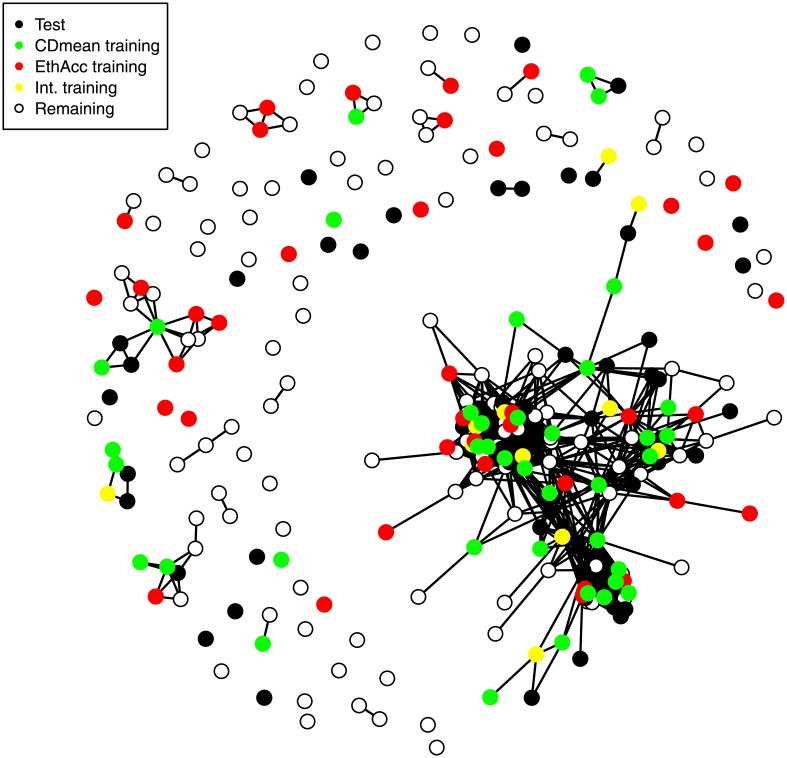
Network representation to highlight the differences in optimized training sets obtained with EthAcc and CDmean as optimization criteria. The data involve a test set of the Flint panel for the DM_Yield trait. This test set had the accuracy of 0.07 and 0.76 when we used as training sets those optimized by CDmean and EthAcc, respectively. The network representation is based on VanRanden’s kinship matrix containing 25 682 SNPs subject to a threshold equal to 0.77 (i.e., individuals were linked in the network if their kinship coefficient was greater than 0.77). Individuals belonging to the test set, the training set optimized via EthAcc, the training set optimized via CDmean, the intersection of the two training sets, and the remaining set are black, red, green, yellow, and white circles, respectively.

To illustrate the difference in optimized training sets when we optimized the trainings using EthAcc or CDmean, we chose the test set that manifested the biggest difference in the TS accuracy values. This test set involved the Flint panel for the DM_Yield trait and a fixed training set size of 50 individuals. Its TS accuracy was 0.07 and 0.76 when CDmean or EthAcc, respectively, served as the optimization criterion. Fig 4 depicts these optimized training sets in a network representation. The kinship matrix that was employed to build the (0-1) network was VanRanden’s kinship matrix subject to a threshold equal to 0.77 (i.e., individuals were linked in the network if their kinship was greater than 0.77).

**Fig 5:**
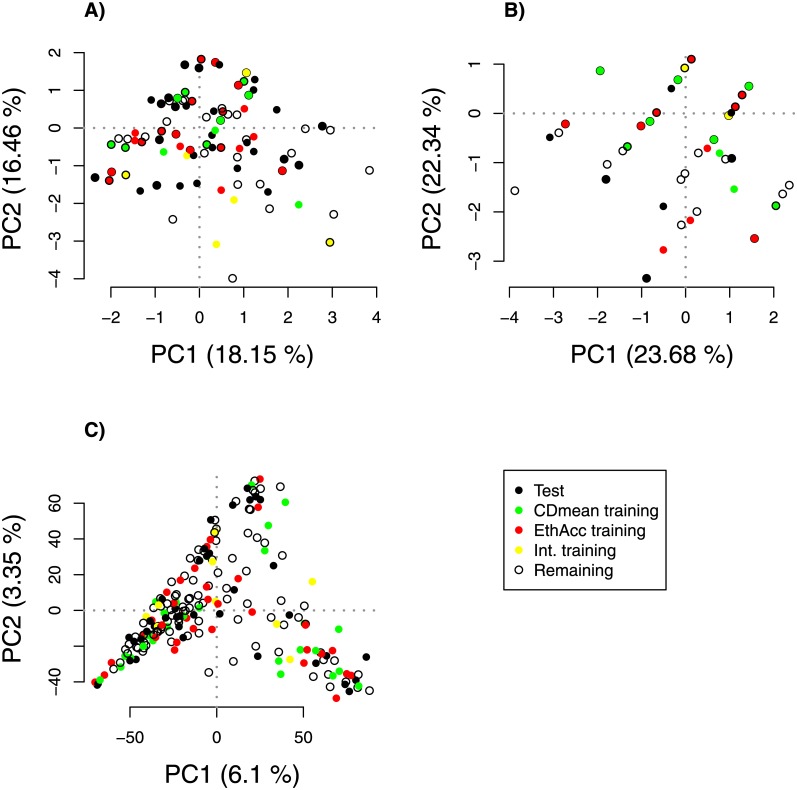
**The first principal component plane based on SNP markers**: A) SNPs detected by the MLMM forward selection approach in the training set optimized by EthAcc, B) SNPs detected by the MLMM forward selection approach in the training set optimized by CDmean, C) all SNPs with MAF greater than 0.05. Test set individuals were projected onto the plane but were not included in the computation of PCA axes. The test set is from the Flint panel for the DM_Yield trait. This test set had the accuracy of 0.07 and 0.76 when we used as training sets those optimized via CDmean and EthAcc, respectively. Individuals belonging to the test set, the training set optimized via EthAcc, the training set optimized via CDmean, the intersection of the two training sets, and the remaining set are plotted as black, red, green, yellow, and white circles, respectively.

Results shown in Fig 4 clearly indicate that the optimization with the EthAcc criterion on the one hand and CDmean criterion on the other hand did not select the same individuals into their training sets to predict the same test set. This difference in selection can explain the extreme difference in the accuracy that was observed with the two training sets. Only 11 individuals out of 50 were identical between EthAcc and CDmean, represented by yellow circles in Fig 4. CDmean chose mostly individuals in the training set that are directly or indirectly linked in the network to individuals in the test set (i.e., kinship coefficient > 0.77). Only three individuals that were less related to the test set were selected. In contrast, EthAcc selected fewer individuals with high kinship coefficients toward test set individuals. Indeed, 18 out of 50 individuals belonging to the training set were unlinked in the network to the individuals of the test set. To delve deeper into what happened, we compared the causal QTLs detected by the forward selection approach of MLMM in the two training sets previously optimized by CDmean and EthAcc. Eight and five SNPs were detected with the EthAcc and CDmean training sets, respectively. Among the eight SNPs detected in the EthAcc training set, a single SNP was rare in the training set (MAF lower than 10% in the training set), whereas three out of five of the SNPs detected in the CDmean were rare (see Tables B and C in S1 File). The reduction in training set diversity caused by the CDmean criterion resulted in GWAS signals for rare SNPs and a prediction with the highest weights on those rare SNPs and on their linked SNPs. To confirm this observation, PCA was conducted with the detected SNPs and with all the SNPs for comparison. All individuals (test, training, and the remaining individuals) were projected onto the first two principal components (Fig 5), but test individuals were not used in the analysis to construct components. We can see that the detected SNPs of the CDmean training set clearly structured the panel by reducing diversity with relatedness, in contrast to the detected SNPs of the EthAcc training set, which did not emphasize any structure.

### Issues with training set optimization via EthAcc

Even though EthAcc performs quite well when the starting set of optimization is the optimal training set, we had to face numerical and algorithmic problems when performing optimization with a random training set as start.

One of these problems was due to what seems to be overparametrization of the step of learning causal QTLs. The causal QTLs were learned with MLMM for each new training set, and some false positive training sets were always produced by the optimization process (i.e., a training set with a high EthAcc value, close to 1, but low TS accuracy). We found a solution to this problem. Indeed, we observed that all false positive training sets yielded a high variance of the TS predicted genetic values. Because this variance is evaluated in the estimated causal-QTL model, it seems that for a given test set, we can always find a training set that produces high dispersion of the TS genetic values. A solution was then to reduce the original phenotype to a variance of one and to prevent EthAcc from reaching these training sets by means of a constraint on this TS genetic value variance (by requiring that it is strictly less than 1). In other words, we forbided EthAcc from estimating a genetic variance greater than the phenotypic variance.

**Fig 6:**
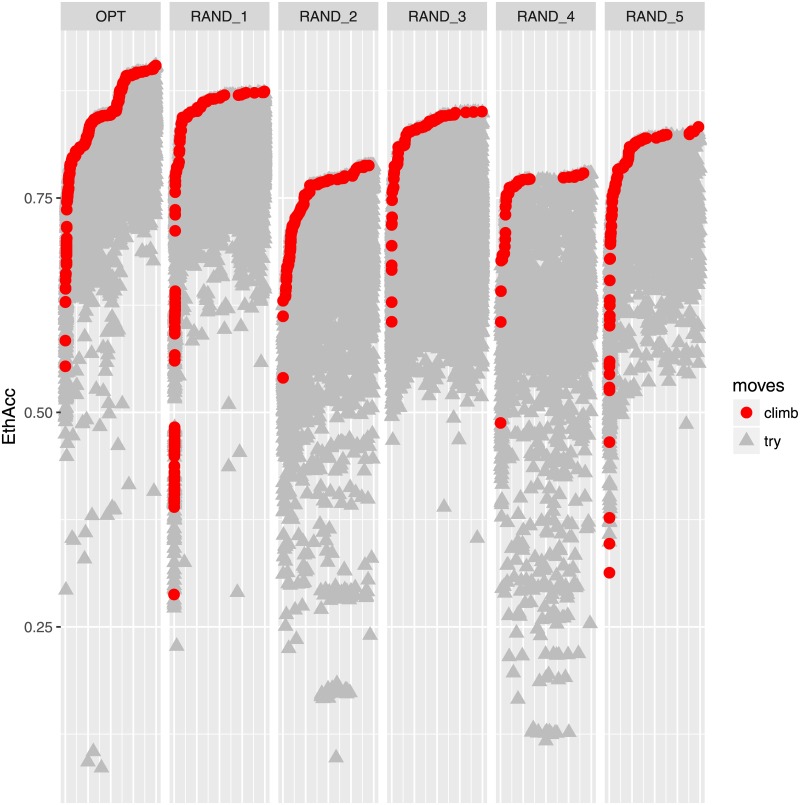
Hill-climbing moves for the optimal (but inaccessible without test phenotypes) training set (first column) and five random training sets serving as starts. Red circles denote the training sets that produced an increase of the current EthAcc value, and grey triangles represent the training sets that did not increase the current EthAcc value. A total of 20 000 moves represented each start.

The second problem is an algorithmic problem, as illustrated in Fig 6. This is a plot of the EthAcc values of training sets computed during the hill-climbing algorithm with 20 000 moves. The same TS was chosen for the optimization process with five random training sets and the optimal one, as starting sets. The hill-climbing moves are represented by red circles and the other moves (except those that are discarded due to the constraint) are plotted as grey triangles. It is clear that despite five random starts, each with 20 000 moves, the maximum value of EthAcc is not yet reached. Finding the training set that maximizes EthAcc is a combinatorial optimization that is really difficult to solve due to the causal-QTL learning step. Other algorithms could have been tested [42]; however, the results would have been almost the same because the optimal training set seems to be very specific.

Moreover, the precision of EthAcc is variable: even though we obtained an average mean square error close to 10^−3^, some training sets yielded an absolute difference between EthAcc and the TS accuracy greater than 0.5. Such a huge difference, due to the QTL estimation step and the simple linear QTL model of the phenotype, prevents the algorithm from reaching obviously desirable training sets: those with both a high EthAcc value and TS accuracy.

## Discussion

We have compared several estimates to infer the accuracy of GBLUP for a given training set (i.e., Pearson’s correlation between the observed phenotype and the predicted genetic value of tests given a training set genotyping). We demonstrated that neither CD- nor PEV-based accuracy estimators are accurate. The reason is that both implied that the causal-QTL model, which emulates the genetic value, is identical to the linear mixed marker model that enables making the prediction. This model equality implies that each marker is a QTL and that the QTL effects are independent and identically distributed according to a Gaussian distribution. These assumptions on QTL effects (and thus on the genetic value) are asymptotically correct in the pedigree mixed model because it is proved to be the consequence of random draws of individuals in a lineage and an infinite number of equal and additive loci [43]. On the other hand, to estimate the accuracy precisely, the essential missing information is the identical-by-descent status of alleles at the causal loci between the test and the training individuals. This missing information has to be reflected by the marker-based kinship matrix, and a lot of research has been published regarding improvement of this kinship estimation [14, 44, 45]. In contrast to this way of thinking stuck in the mixed model framework, Rabier et al. [20] proposed an estimate of the accuracy by working in an instrumental mixed marker model to predict the genetic value and a causal fixed linear model to emulate the genetic values. Into their theoretical accuracy formula, we plugged the location and the SNP effect estimated by the forward MLMM approach [28]. Thus, we showed on real data that we obtained an accurate estimate of the accuracy (MSE of 10^−3^ on average among sugar beet traits).

Having an estimate of the accuracy for a given training set requires knowledge of the genotyping of the training set but allows researchers to easily obtain an estimate of the expectation of the accuracy for a training set randomly drawn in a population. Indeed, this accuracy expectation can be calculated by performing random draws of training sets and taking the mean. This accuracy expectation was the goal of the different formulas derived from the works of [15, 16]. Via estimation of the QTL locations and effects, the authors of [27] compared the expectation of the theoretical accuracy to these analytical formulas and found that the former performed better than the other formulas. The most important issue with the theoretical accuracy formula is the estimation of the causal-QTL locations and effects. The authors of [27] compared different penalized regression methods and revealed that adaptive LASSO [32] performed the best. We also compared different methods by plugging them into the theoretical accuracy formula, but in contrast to [27], we did not use the penalized regression estimators of the QTL effects because they are known to be biased. We used a two-step procedure to avoid this bias, whereas MLMM and penalized regression methods were employed to locate the QTLs, and their effects were estimated by classical ordinary least square methods. We based our choice to locate the QTLs on the comparison implemented by [30] for GWAS, and we added the analytical FDR control because it has been shown to perform the best on SNP selection [31], MLMM [28] and adaptive LASSO [32]. We ranked EN (except with the FDR control) before LASSO in terms of mean squared error as compared to the TS accuracy. Adaptive LASSO was ranked after penalized regressions that based their choice for the LASSO skrinkage parameter on the criterion of minimum MSE plus one standard error; however, MLMM was the best performer.

Accordingly, the theoretical accuracy with QTL locations obtained by MLMM was named EthAcc for Estimated THeoretical ACCuracy. We proposed R code to compute EthAcc that does not involve Henderson’s mixed model equations, as opposed to [23] and [33], but made all the calculations based on the ridge regression matrix $H=\left(X_{\text{train}}X_{\text{train}}^{\prime }+\frac{\sigma_{\varepsilon }^{2}}{\sigma_{\beta }^{2}}I\right)$, where ***X***_train_ is a matrix of genomic information of training individuals. The reason is the necessity to have an invertible kinship matrix to use Henderson’s equations, which is not the case for VanRaden’s kinship matrix. When not invertible, the kinship matrix is generally projected on the cone of positive–definite matrices which create computation approximations. Moreover, Henderson’s equations are effective when the size of recorded data is huge as compared to the training set size, which is generally the case in animal breeding evaluation as the data are recorded on females while the males are evaluated. By contrast, in plant breeding, there is generally one record per individual on evaluation; therefore, it is simple and more effective to use ridge regression matrix ***H***. Nevertheless, EthAcc can also be computed via Henderson’s equations; this approach makes sense when the kinship matrix is not a marker-based matrix.

EthAcc allows us to perform optimization of training individuals to predict and we compared it to training set optimization with CDmean [23]. EthAcc clearly outperformed CDmean on all the real data we tested (sugar beet, maize, and wheat). Nonetheless, this performance was due to the fact that we started the optimization burden with the optimal training set by means of the test set phenotypes to find it. Close to this optimal training set, EthAcc finds a good training set (because it is an accurate estimate of the accuracy), whereas CDmean chose a training set that strongly decreases the maximum of the TS accuracy by increasing the relatedness of individuals. In contrast, with a random training set as start, algorithmic and precision issues prevent EthAcc from reaching good training sets and despite a lot of attempts we did not succeed in making EthAcc perform correct training set optimization. Certainly, we could have performed optimization with a more effective algorithm than the hill-climbing algorithm or we could have increased the number of hill-climbing moves. MLMM is a long CPU software application, especially with a high number of markers but it could be replaced by the best penalized regression which is much faster and therefore allows much more moves. Nevertheless, the crucial point is the precision of EthAcc. Indeed, a MSE of 10^−3^ on average is not a guarantee of having a close estimate of the accuracy. During the optimization process, some training sets were mostly overestimated then yielding false positive training sets. The precision of the theoretical accuracy—with known causal-QTL locations and effects—was shown to be much higher [20, 27]. This finding suggests that the QTL localization should be improved. Adding functional information such as candidate genes, metabolic networks, and transcription factors may help to explore various ways to facilitate the QTL localization by limiting the search to fragments of the genome that are known to be involved in the trait of interest. Nevertheless, the task of funding causal genome fragments is daunting, and if there are too many missing causal regions, a decrease in accuracy can be observed, as in sunflower hybrids when the oil metabolic network is used to predict oil content [36]. EthAcc is derived from a linear additive causal-QTL model. This simple model can be made more real by modeling the interaction between causal QTLs. A more generalized theoretical formula has to be devised in such a model, but the procedure developed in [20] can be applied without any difficulty.

Despite the drawbacks of EthAcc, we illustrated that a substantial gain in accuracy can be obtained by performing training set optimization (up to 100% in the Flint maize panel with a yield). This accomplishment is quite different from the small differences among all the published methods for improving prediction (i.e., linear mixed models, penalized regression analyses, Bayesian methods, and nonparametric models). Such an increase in accuracy is worth the time spent on work on training set optimization, in particular with a complex and highly polymorphic trait. Moreover, we showed that increasing the training population size does not always lead to better performance relative to optimization of the training set.

## Supporting information

S1 File Content. EthAcc R code; Sugar beet material in details; Standard error correction to take into account the dependency of test sets generated by the sampling process; (Fig A): First principal component plane of the sugar beet panel using 836 SNP markers and showing the structure of the panel in two clusters; Table A: P-value of the significance test of difference between the TS accuracy and that estimated by EthAcc, CD and PEV using sugar beet structures in two clusters (Panel_A and Panel_B) on several traits (100 random test sets); Table B: MAF of SNPs detected using MLMM in the training set optimized via EthAcc and Table C: MAF of SNPs detected using MLMM in the training set optimized via CDmean.
(PDF)
