# Supplementary material for: Training set optimization of genomic prediction by means of EthAcc
Source: PLoS One. 2019 Feb 19;14(2):e0205629. doi: 10.1371/journal.pone.0205629 (PMC6380617; doi:10.1371/journal.pone.0205629)
Supplement: S1 File — EthAcc R code; Sugar beet material in details; Standard error correction to take into account the dependency of test sets generated by the sampling process; (Fig A): First principal component plane of the sugar beet panel using 836 SNP markers and showing the structure of the panel in two clusters; Table A: P-value of the significance test of difference between the TS accuracy and that estimated by EthAcc, CD and PEV using sugar beet structures in two clusters (Panel_A and Panel_B) on several traits (100 random test sets); Table B: MAF of SNPs detected using MLMM in the training set optimized via EthAcc and Table C: MAF of SNPs detected using MLMM in the training set optimized via CDmean. (PDF) [file pone.0205629.s001.pdf]

EthAcc R code

```
#########################################################
# note: require rrBLUP, glmnet, parcor, EN.FDR.r and mlmm.gwas
# Function that computes the Estimated THeoritical ACCuracy (EthAcc)
# on the basis of the formula of Rabier et al. PlosOne, 2016
# Causal SNPs can be located by several gwas methods or given by the user
#Entry
#-----
#x_train is the SNP dose matrix for the training population. It is a n by m matrix,
#               where n=number of  individuals, m=number of SNPs,
#               with rownames(x_train)=individual names, and colnames(x_train)=SNP names.
#x_test is the same codage matrix as x_train but for individuals in the test population
#y_train is the phenotype of individual in the training population.
#               It is a vector of length n=number of  individuals,
#               with names(y_train)=individual names
#snp.pop_train is the name of the SNPs=QTLs in the causal model if known,
#               must be included in colnames(x_train)
#meth is the method to find causal SNPs, can be "MLMM", "EN05.FDR", "adpLASSO"
#               or a triple for penalized method with
#         alpha value, "min" or "1se", TRUE or FALSE for SNP standardization
#examples:
#res.EthAcc<-compute.EthAcc(x.train,x.test,y.train,snp.pop_train=colnames(x.train)[1:10])
#res.EthAcc<-compute.EthAcc(x.train,x.test,y.train,meth="MLMM")
#res.EthAcc<-compute.EthAcc(x.train,x.test,y.train,meth=c(0.5,"1se",TRUE) )
#
#WARNING: too small MAFs in x.train give innacurate results
#################
###############auxiliary functions
library(rrBLUP)  #dependency on rrBLUP package
library(mlmm.gwas) #dependency on mlmm.gwas package
source("EN.FDR.r")
library(glmnet) #dependency on glmnet
library(parcor) #dependency on parcor
############################################
# function to compute VanRanden type kinship
kinship<-function(x){
  x.center<-scale(x,center=TRUE,scale=FALSE)
  KK<-x.center%*%t(x.center)
  cst.VR<-sum(apply(x.center,2,var))
  KK<-KK/cst.VR
  KK
  return(list(KK=KK, cst.VR=cst.VR)) #kinship and VanRandem constant
}
#function to find causal QTL
gwas.togetcausalSNP<-function(x_train,y_train,meth){
  snp.pop_train<-NULL
  x_train.c<-scale(x_train,center=TRUE,scale=FALSE)
  nstep<-length(y_train) -10  #10 to keep degrees of freedom for the residual
  if(length(meth)==1){
    if(meth=="MLMM"){
      kk.pop_train<-kinship(x_train)$KK  #kinship for GWAS
      gwas.pop_train<-mlmm_allmodels(y_train,list(x_train),list(kk.pop_train),2,nstep)
      snp.pop_train<-NomSNP(gwas.pop_train) #estimated causal SNP
    }
```

```
    if(meth=="EN05.FDR"){ #Yi et al Genetics 2015
      res.enfdr<-EN.FDR(NULL,x_train,y_train,0.5,1000,0.05)
      snp.pop_train<-names(res.enfdr$betas.SNP)
    }
    if(meth=="adpLASSO"){ #adaptive LASSO
      x_train.cr<-scale(x_train,center=TRUE,scale=TRUE)
      y_train.cr<-scale(y_train,center=TRUE,scale=TRUE)
      res.adpLASSO<-adalasso(x_train.cr, y_train.cr,k=5)
      snp.pop_train<-colnames(x_train)[res.adpLASSO$coefficients.adalasso!=0]
    }
  }
  if(length(meth)==3){
    if(meth[3]==TRUE) {  #penalized regression
      res.lasso<-glmnet(x_train.c,y_train,family="gaussian",standardize=TRUE,
alpha=as.numeric(meth[1]))
      res.cv.lasso<-cv.glmnet(x_train.c,y_train,family="gaussian",standardize=TRUE,
alpha=as.numeric(meth[1]))
    }
    if(meth[3]==FALSE) {
      res.lasso<-glmnet(x_train.c,y_train,family="gaussian",standardize=FALSE,
alpha=as.numeric(meth[1]))
      res.cv.lasso<-cv.glmnet(x_train.c,y_train,family="gaussian",standardize=FALSE,
alpha=as.numeric(meth[1]))
    }
    if(meth[2]=="min"){
      temp<-abs(res.lasso$lambda-res.cv.lasso$lambda.min)
      id.lambda<-which(temp==min(temp))
      snp.pop_train<-names(which(res.lasso$beta[,id.lambda]!=0))
    }
    if(meth[2]=="1se"){
      temp<-abs(res.lasso$lambda-res.cv.lasso$lambda.1se)
      id.lambda<-which(temp==min(temp))
      snp.pop_train<-names(which(res.lasso$beta[,id.lambda]!=0))
    }
  }
  snp.pop_train
}
# function to get associated SNP in mlmm results
NomSNP<-function(res.mlmm){
  names.snp<-NULL
  last.snp<-NULL
  n.step<-length(res.mlmm)
  if(n.step>2) {
    id<-grep("selec_",names(res.mlmm[[n.step]]) )
    names.snp<-names(res.mlmm[[n.step]])[id]
      names.snp<-unlist(sapply(names.snp,function(x){
      unlist(strsplit(x,"selec_"))[2]
      }))
   #add the last associated SNP
   id<-which( res.mlmm[[n.step]][-(1:(n.step-2))]==min( res.mlmm[[n.step]][-(1:(n.step-2))],
na.rm=TRUE ) )
   last.snp<-names( res.mlmm[[n.step]])[-(1:(n.step-2))][id]
  }
  if(n.step==2) {
    id<-which(res.mlmm[[n.step]]==min( res.mlmm[[n.step]] ,na.rm=TRUE) )
    last.snp<-names( res.mlmm[[n.step]])[id]
```

```
  }
  names.snp<-c(names.snp,last.snp)
}
# function to compute theoretical accuracy
Theo.acc<-function(Mtest,Mtrain,effect,Hinv,Ve){
#theoritical formula of Rabier et al. PlosOne, 2016, adapted to non centered genotypic matrices
  if( is.null(effect) ) return(NA)
  ##sort  on names
  if( length(effect) > 1 ) { effect<-effect[sort(names(effect))] }
  Mtrain <- Mtrain[,sort(colnames(Mtrain))]
  Mtest <- Mtest[,sort(colnames(Mtest))]
  if( length(effect) > 1 ) {
    #predictor for training individuals
    predtrain <- Mtrain[,which(colnames(Mtrain)%in%names(effect))] %*% as.matrix(effect)
    #predictor for test individuals
    predtest <- Mtest[,which(colnames(Mtest)%in%names(effect))] %*% as.matrix(effect)
  } else {
    #predictor for training individuals
    predtrain <- as.matrix( Mtrain[,which(colnames(Mtrain)%in%names(effect))] * effect )
    #predictor for test individuals
    predtest <- as.matrix( Mtest[,which(colnames(Mtest)%in%names(effect))] * effect )
  }
  Mtest<-scale(Mtest,center=TRUE,scale=FALSE)  #case of non centered Mtest
  mu.Hinv<- as.matrix(apply(Hinv,1,sum)) #case of non centered Mtrain
  Hinv.cor<- Hinv -  ( mu.Hinv %*% t(mu.Hinv)) / sum(Hinv) #case of non centered Mtrain
  espfortrain <- t(Mtrain) %*% Hinv.cor %*% predtrain
  nume <- t(predtest) %*% Mtest %*% espfortrain / nrow(Mtest)
  RROracle <- Mtest %*% t(Mtrain) %*% Hinv.cor
  termDesign <- Ve * sum(RROracle^2)/ nrow(Mtest)
  termvar <- t(espfortrain) %*%  t(Mtest) %*% Mtest  %*% espfortrain /nrow(Mtest)
  Vg <- var( predtest) #genetic variance in the causal model estimated on test individuals
  if( Vg >1) print("WARNING: estimated genetic variance too great, result innacurate")
  res <- nume / sqrt( ( termDesign + termvar) * (Vg + Ve) )
  res
}
#################principal function
compute.EthAcc<-function(x_train,x_test,y_train,snp.pop_train=NULL,meth=NULL){
#controls
 if(is.null(snp.pop_train) & is.null(meth) ) stop
 stopifnot( ncol(x_train)==ncol(x_test) )
 stopifnot( length(y_train)==nrow(x_train) )
 x_train<-x_train[,sort(colnames(x_train))]
 x_test<-x_test[,sort(colnames(x_test))]
 stopifnot( sum( colnames(x_train)!=colnames(x_test) )==0 )
 if(!is.null(snp.pop_train)) stopifnot( length( which( colnames(x_train)%in%snp.pop_train) ) ==
length(snp.pop_train) )
 x_train<-x_train[sort(rownames(x_train)),]
 y_train<-y_train[sort(names(y_train))]
 stopifnot( sum( rownames(x_train)!=names(y_train) )==0 )
#estimate causal location by gwas
 if(is.null(snp.pop_train)) snp.pop_train<-gwas.togetcausalSNP(x_train,y_train,meth)
#estimation
 y_train<-y_train/sd(y_train)  #standardization of phenotype
 #get Hinv in rrBLUP model
 rrblup.pop_train<-mixed.solve(y_train,X= rep(1,length(y_train)),Z=x_train,K=NULL,SE=FALSE,
return.Hinv=TRUE)
```

```
x.snp.pop_train<-as.matrix(x_train[, snp.pop_train ])
  if(length(snp.pop_train)<(length(y_train)-1) & length(snp.pop_train)!=0){
    lm.in.causal<-lm(y_train~1+x.snp.pop_train) #causal model
    eff.snp.pop_train<-lm.in.causal$coefficients[-1] #causal SNP=QTL effect estimation
    names(eff.snp.pop_train)<-snp.pop_train
    eff.snp.pop_train<-eff.snp.pop_train[!is.na(eff.snp.pop_train)]
#residual variance estimation in the causal model
    ve.pop_train<-summary(lm.in.causal)$sigma^2
    res<-Theo.acc(x_test,x_train,eff.snp.pop_train,rrblup.pop_train$Hinv,ve.pop_train)
  } else {res<-NA}
names(res)<-paste("EthAcc", do.call(paste, c(as.list(meth), sep="_")),sep='_' )
res
}
```

Sugar beet material in details

Panels A panel of 2101 elite lines of diploid sugar beet (*Beta vulgaris* L.), which resulted from many different crosses in Florimond Desprez's breeding program, was analyzed in this study. This panel represented the pollinator pool that was evaluated in testcrosses in the company multienvironment trials (MET) in 2009, 2010 and 2011. Testcross progenies were produced by crossing each elite line to the same single-cross hybrid as a tester.

Phenotypic data The 2101 testcross progenies were evaluated in unbalanced MET. In 2009, 765 progenies were phenotyped in 24 different locations however each progeny was evaluated in six to nine locations only. In 2010, 742 individuals were phenotyped in 12 different locations (from 5 to 8 per progeny), among them 4 were also phenotyped in 2009. Finally, 618 progenies were phenotyped in 2011 in 32 different locations. Each progeny was evaluated in five to ten locations and 20 individuals were also phenotyped in 2010. Two control varieties were common between all years and locations. The 7 evaluated traits were: potassium content (K, meq/100g) measured by a flame photometer, sodium content (Na, meq/100g) measured by a flame photometer, α-amino nitrogen content (N, meq/100g) measured by colorimetry, sugar content (S, %) measured by polarimetry, the root yield (RY, t/ha), white sugar content (WS, %) calculated as S - (0.14 x (K + Na) + 0.25 x N + 0.5) and finally the white sugar yield (WSY, t/ha) calculated as (RY x WS) / 100.

Phenotypic data analysis Trait data were analyzed using a two-stage analysis in R [1]. The first stage was dedicated to the analysis of the different traits in single environment according to the experimental alpha designs that were set up, producing reliable adjusted phenotypes per environment. These adjusted phenotypes were calculated with a linear mixed model by fitting a complete block effect as fixed, whereas row, columns and genetic effects were modeled as independent random effects. The following linear mixed model was then used to estimate variance components of the testcrosses and to get average phenotype: $y_{ij} = \mu + env_i + G_j + \epsilon_{ij}$, where y_{ij} is the adjusted phenotype of the jth sugar beet line in the ith environment, μ the global mean , env_i the effect of the ith environment, G_j the genetic effect of the jth sugar beet line, and ϵ_{ij} the residual term including the genotype by environment interaction effect. Environment and genetic effects were modeled respectively as fixed and random independent effects. From this model, the average phenotype of each testcross was computed as $\hat{\mu} + \hat{G}_i$. These average phenotypes were used as the observed phenotypes for the genomic prediction study.

Genotypic data The 2101 breeding panel lines were fingerprinted with 836 SNP markers. The markers used in this study were designed in both genic and intergenic sequences (cDNAs) in a set of elite lines and had previously been mapped using three different F2 mapping populations, as described by [2]. The length of the total genetic map is 705 cM, with chromosome sizes estimating between 70 cM and 91 cM for chromosome 5 and chromosome 3, respectively. The samples used for DNA fingerprinting profiles were leaves of one plant per breeding line. Leaf disks were sampled, frozen at -80°C and freeze-dried. DNA extraction was performed using the NucleoSpin® Plant kit (Machery-Nagel, Düren, Germany) and genotyping was performed for individual SNPs using KASP genotyping chemistry (LGC Genomics, Teddington Middlesex, United-Kingdom).

Among the SNP markers, markers were filtered on their minimum allele frequency (MAF) (greater than 2%) and on percentage of missing data (less than 15%). This SNP selection yielded a total of 692 SNP markers that were employed for the genomic selection analysis. Imputation of missing marker genotypes was done by the mean genotypic value.

Panel structure The structure of subpopulations in this panel was also studied. We applied hierarchical clustering to principal components using the FactoMineR package `https://cran.r-project.org/web/packages/FactoMineR/index.html` [3] in R software to assign each individual to a subpopulation after principal component analysis (PCA). The HCPC function of the FactoMineR package implements this calculation after having constructed the hierarchy and suggests an optimal level for division (Fig A).

Standard error correction to take into account the dependency of test sets generated by the sampling process

It is important to test if an estimator of the accuracy is significantly different to the TS accuracy, but the lack of independence between the sampled test sets makes it hard to obtain a correct estimate of the variance of the mean difference. This variance is necessary to build a test of significance. Neither the division by the square root of the number of sampled test sets nor the bootstrapped variance are correct with dependent results. Both methods provide a too small variance of the mean difference and thus conclude significance whereas there is no significance. Nonetheless, [4] proposed a correction of the standard deviation of the mean difference that allows to build a test that performs correctly both in term of type one error and power. This correction takes into account the average of overlap information between two random test sets. Let p_{test} be the proportion of sampled test individuals in the whole population, the variance of the mean difference is multiply by $\sqrt{1/n_{TS} + p_{test}/(1-p_{test})}$, instead of $\sqrt{1/n_{TS}}$ when samples are independent, where n_{TS} is the number of sampled test sets.

References

[1] R Core Team. R: A Language and Environment for Statistical Computing; 2015. Available from: `https://www.R-project.org`.

[2] Adetunji I, Willems G, Tschoep H, Bürkholz A, Barnes S, Boer M, et al. Genetic diversity and linkage disequilibrium analysis in elite sugar beet breeding lines and wild beet accessions. Theoretical and applied genetics. 2014;127(3):559–571.

[3] Lê S, Josse J, Husson F, et al. FactoMineR: an R package for multivariate analysis. Journal of statistical software. 2008;25(1):1–18.

[4] Nadeau C, Bengio Y. Inference for the generalization error. In: Advances in neural information processing systems; 2000. p. 307–313.

Fig A: First principal component plane of the sugar beet panel using 836 SNP markers and showing the structure of the panel in two groups.

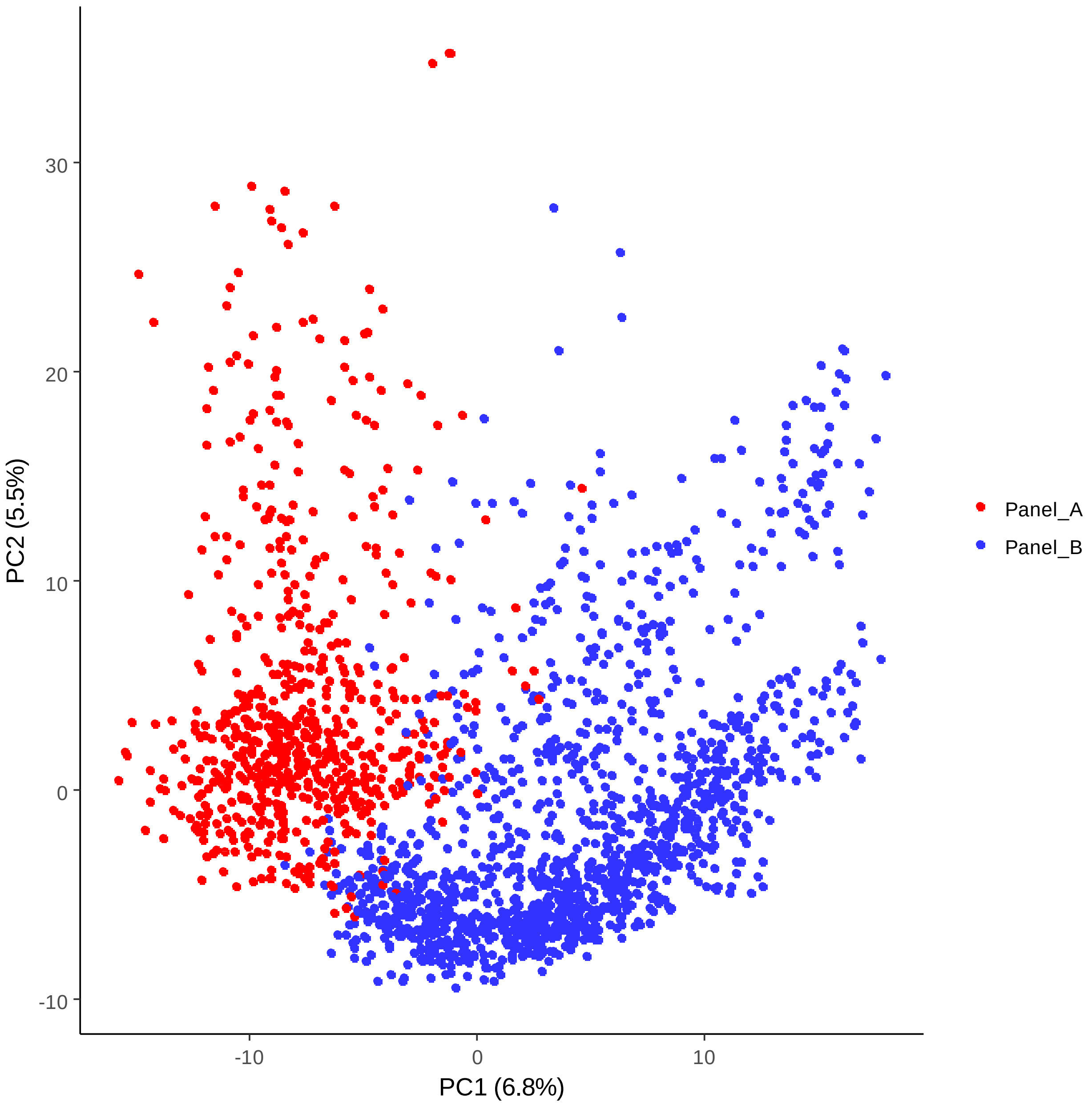

Table A: P-value of the significance test of difference between the TS accuracy and that estimated by EthAcc, CD and PEV using sugar beet structures in two clusters (Panel_A and Panel_B) on several traits (100 random test sets).

Trait[a]	Test set[b]	Training set[b]	P-value		
			EthAcc	CD	PEV
K	Panel_A	Panel_A+B	$4.59\ 10^{-01}$	$9.46\ 10^{-06}$	$8.73\ 10^{-06}$
K	Panel_A	Panel_A	$6.99\ 10^{-01}$	$1.66\ 10^{-04}$	$1.17\ 10^{-04}$
Na	Panel_A	Panel_A+B	$5.41\ 10^{-01}$	$1.42\ 10^{-09}$	$1.35\ 10^{-09}$
Na	Panel_A	Panel_A	$6.07\ 10^{-01}$	$7.02\ 10^{-04}$	$5.74\ 10^{-04}$
N	Panel_A	Panel_A+B	$9.41\ 10^{-02}$	$1.29\ 10^{-14}$	$1.16\ 10^{-14}$
N	Panel_A	Panel_A	$6.39\ 10^{-01}$	$3.56\ 10^{-03}$	$3.06\ 10^{-03}$
SC	Panel_A	Panel_A+B	$4.04\ 10^{-01}$	$1.80\ 10^{-10}$	$1.67\ 10^{-10}$
SC	Panel_A	Panel_A	$7.61\ 10^{-01}$	$1.24\ 10^{-05}$	$9.50\ 10^{-06}$
WSC	Panel_A	Panel_A+B	$6.63\ 10^{-01}$	$7.22\ 10^{-09}$	$6.78\ 10^{-09}$
WSC	Panel_A	Panel_A	$9.56\ 10^{-01}$	$1.56\ 10^{-04}$	$1.23\ 10^{-04}$
RY	Panel_A	Panel_A+B	$9.60\ 10^{-01}$	$1.64\ 10^{-08}$	$1.55\ 10^{-08}$
RY	Panel_A	Panel_A	$7.46\ 10^{-01}$	$4.21\ 10^{-03}$	$3.63\ 10^{-03}$
WSY	Panel_A	Panel_A+B	$5.55\ 10^{-01}$	$1.33\ 10^{-05}$	$1.28\ 10^{-05}$
WSY	Panel_A	Panel_A	$8.97\ 10^{-01}$	$4.44\ 10^{-02}$	$4.13\ 10^{-02}$
K	Panel_B	Panel_A+B	$1.19\ 10^{-01}$	$8.18\ 10^{-12}$	$7.\ 01\ 10^{-12}$
K	Panel_B	Panel_B	$9.33\ 10^{-01}$	$2.47\ 10^{-04}$	$1.93\ 10^{-04}$
Na	Panel_B	Panel_A+B	$2.23\ 10^{-01}$	$8.78\ 10^{-13}$	$7.14\ 10^{-13}$
Na	Panel_B	Panel_B	$7.62\ 10^{-01}$	$5.31\ 10^{-03}$	$3.93\ 10^{-03}$
N	Panel_B	Panel_A+B	$9.79\ 10^{-02}$	$1.66\ 10^{-21}$	$1.44\ 10^{-21}$
N	Panel_B	Panel_B	$7.54\ 10^{-01}$	$3.34\ 10^{-12}$	$2.51\ 10^{-12}$
S	Panel_B	Panel_A+B	$2.64\ 10^{-01}$	$4.00\ 10^{-14}$	$3.15\ 10^{-14}$
S	Panel_B	Panel_B	$9.12\ 10^{-01}$	$2.22\ 10^{-04}$	$1.62\ 10^{-04}$
WS	Panel_B	Panel_A+B	$2.62\ 10^{-01}$	$4.29\ 10^{-14}$	$3.63\ 10^{-14}$
WS	Panel_B	Panel_B	$7.39\ 10^{-01}$	$2.12\ 10^{-04}$	$1.61\ 10^{-04}$
RY	Panel_B	Panel_A+B	$6.51\ 10^{-02}$	$2.87\ 10^{-13}$	$2.61\ 10^{-13}$
RY	Panel_B	Panel_B	$7.11\ 10^{-01}$	$6.90\ 10^{-05}$	$5.29\ 10^{-05}$
WSY	Panel_B	Panel_A+B	$8.45\ 10^{-01}$	$1.82\ 10^{-16}$	$1.67\ 10^{-16}$
WSY	Panel_B	Panel_B	$7.53\ 10^{-01}$	$5.83\ 10^{-07}$	$4.58\ 10^{-07}$
K	Panel_A+B	Panel_A+B	$9.93\ 10^{-01}$	$2.63\ 10^{-11}$	$1.72\ 10^{-11}$
Na	Panel_A+B	Panel_A+B	$9.13\ 10^{-01}$	$8.96\ 10^{-06}$	$6.65\ 10^{-06}$
N	Panel_A+B	Panel_A+B	$9.35\ 10^{-01}$	$8.75\ 10^{-14}$	$7.07\ 10^{-14}$
S	Panel_A+B	Panel_A+B	$4.05\ 10^{-01}$	$1.13\ 10^{-13}$	$7.71\ 10^{-14}$
WS	Panel_A+B	Panel_A+B	$8.79\ 10^{-01}$	$2.28\ 10^{-13}$	$1.59\ 10^{-13}$
RY	Panel_A+B	Panel_A+B	$9.42\ 10^{-01}$	$7.03\ 10^{-15}$	$5.02\ 10^{-15}$
WSY	Panel_A+B	Panel_A+B	$8.75\ 10^{-01}$	$1.70\ 10^{-08}$	$1.40\ 10^{-08}$

[a] potassium content in meq/100g (K), sodium content in meq/100g (NA), α-amino nitrogen content in meq/100g (N), sugar content in % (S), white sugar content in % (WS), the root yield in t/ha (RY), the white sugar yield in t/ha (WSY)

[b] cluster(s) to which the individual belongs

Table B: MAF of SNPs detected using MLMM with the training set chosen via EthAcc. MAF is calculated for the training set, the test set and the candidate set. Results concern the Flint panel for DM_Yield trait. The test set had the accuracy of 0.07 and 0.76 when using as training sets those optimized via CDmean and EthAcc, respectively.

SNP	training set	test set	candidate set
PZE.101093639	0.10	0.06	0.10
PZE.102125621	0.39	0.35	0.37
PZE.103139617	0.48	0.39	0.44
PZE.104026198	0.14	0.21	0.12
PZE.104040856	0.35	0.37	0.41
PZE.105054217	0.33	0.37	0.33
PZE.105161112	0.21	0.33	0.25
PZE.107053604	0.42	0.35	0.42

Table C: MAF of SNPs detected using MLMM with the training set chosen via CDmean. MAF is calculated for the training set, the test set and the candidate set. Results concern the Flint panel for DM_Yield trait. The test set had the accuracy of 0.07 and 0.76 when using as training sets those optimized via CDmean and EthAcc, respectively.

SNP	training set	test set	candidate set
PZE.101149675	0.02	0.06	0.07
PZE.101221278	0.10	0.10	0.15
PZE.103073990	0.08	0.12	0.13
PZE.105049283	0.38	0.26	0.42
PZE.110050786	0.32	0.35	0.28
